# In-clinic versus at-home gait kinematics in adults with and without stroke: Associations across environments

**DOI:** 10.1371/journal.pone.0361481

**Published:** 2026-11-11

**Authors:** Rebecca Ahrns, Ryan T. Roemmich, Jan Stenum, Kendra M. Cherry-Allen

**Affiliations:** 1 Department of Physical Therapy Education, Western University of Health Sciences, College of Health Sciences—Northwest, LebanonOregon, United States of America; 2 Department of Physical Medicine and Rehabilitation, Johns Hopkins University School of Medicine, Baltimore, Maryland, United States of America; 3 Center for Movement Studies, Kennedy Krieger Institute, Baltimore, Maryland, United States of America; Pennsylvania State University Main Campus: The Pennsylvania State University - University Park Campus, UNITED STATES OF AMERICA

## Abstract

**Importance:**

Stroke remains a leading cause of mortality and long-term disability in the United States. Gait dysfunction following stroke can limit mobility, participation, and quality of life, even among individuals deemed capable of community ambulation. Gait is commonly assessed in clinical settings under the assumption that it reflects real-world gait; however, empirical support for this assumption remains limited.

**Objective:**

To determine whether gait kinematics measured in a clinical setting reflect gait kinematics measured in real-world environments.

**Design:**

Smartphone or tablet video recordings of participants walking in both clinic and home environments were collected and processed using pose estimation and MATLAB-based analysis to extract gait kinematics. Data from 43 participants (18 with history of stroke, 25 without history of stroke), were analyzed.

**Measures:**

Gait kinematic variables derived from video-based pose estimation, including step length asymmetry (SLA), step time asymmetry (STA), and gait speed (GS).

**Results:**

Spearman correlation analyses revealed weak-to-strong associations between in-clinic and at-home gait metrics with moderate-to-strong correlations among participants with history of stroke (SLA: 𝜌 = 0.455, p = 0.058; STA: 𝜌 = 0.519, p = 0.027; GS 𝜌 = 0.752, p=<0.001) and weak-to-moderate correlations among participants without history of stroke (SLA: 𝜌 = 0.116, p = 0.580; STA: 𝜌 = 0.406, p = 0.044; GS: 𝜌 = 0.541, p = 0.005). Wilcoxon signed-rank analyses indicated no statistically significant differences between most gait metrics between settings (α < 0.05), except STA among participants without history of stroke (p = 0.01).

**Conclusions:**

These findings support the feasibility of using smartphone- or tablet-based video with pose estimation to assess real-world gait. Additionally, they suggest that clinic- and home-based gait are related but not identical. While several gait parameters demonstrated moderate-to-strong correlations between environments, others exhibited weak associations. Together, these findings indicate that clinic-based assessments may provide useful information regarding certain aspects of real-world gait but may not fully represent gait in the home environment.

## Introduction

Stroke remains a leading cause of death and long-term disability in the United States [1–3]. The number of individuals with a history of stroke is projected to rise from 10.8 million in 2025 to 14.5 million by 2060 [4]. Only 32–47 percent of people with a history of stroke can ambulate independently in their communities [5], and many do so with asymmetric gait mechanics [6]. Gait deviations following stroke include temporal asymmetry, spatial asymmetry, and decreased gait speed [7]. Gait dysfunction after stroke limits functional mobility and participation, ultimately reducing quality of life [8]. Additionally, gait dysfunction after stroke can increase risk of falling, which may lead to injury and subsequent hospitalization. These events can further restrict an individual’s mobility, resulting in a cycle that reinforces reduced walking ability [9]. Thus, restoring gait is a primary goal of post-stroke rehabilitation [8–10].

Post-stroke gait dysfunctions—including slowed gait speed and gait asymmetries—have been shown to worsen over time following discharge from rehabilitation, highlighting the importance of follow-up and longitudinal assessment of mobility within a person’s natural environment [6]. Furthermore, capturing gait metrics that reflect how people walk in the real world is essential for developing interventions that truly restore mobility after stroke. Standard clinical assessments, typically conducted in controlled settings, are commonly used to estimate walking ability [10]. These tests measure how an individual performs under ideal conditions and require administration by a trained clinician. For example, the 6-Minute Walk Test and the 10-Meter Walk Test measure what individuals *can* do in the clinic rather than what they *actually* do in their daily environment [5,11]. Consequently, a person may demonstrate the capability to ambulate in the community during testing yet remain inactive once home. More detailed gait information at the impairment level (e.g., joint kinematics, spatiotemporal gait parameters) can be obtained using three-dimensional motion capture systems, but these approaches are expensive and limited to specialized laboratory environments [12]. Thus, there is a critical need for assessment tools that are low-cost, accessible, and capable of objectively capturing how individuals move in real-world settings.

Advanced technologies have recently emerged, enabling researchers to gather quantitative data of movement in any setting [13,14]. Video-based pose estimation [15] has the potential to allow researchers and clinicians to obtain objective data of movement analyses offering the possibility for more accessible quantitative assessment of movement including exciting prospects for telerehabilitation [11]. Pose estimation used in this study identifies anatomical landmarks on a person’s image within each frame of two-dimensional video obtained from a smartphone or tablet camera [16].

This technology has been used to study different movements in a variety of populations as outlined in Stenum and colleagues’ 2021 study [17]. For example, pose estimation has explored a wide range of healthy adult movements, including gait, and has been used to assess movement in populations with neurologic diagnoses including Parkinson’s disease, cerebral palsy, stroke, and dementia [17]. This technology can provide accurate overground gait metrics without access to a motion capture system in both healthy adults and adults with neurological disorders creating the opportunity to discover whether gait kinematics observed in clinical settings reflect those exhibited in individuals’ everyday environments [13,16].

We leveraged freely available pose estimation [15] technology to capture real-world gait metrics in people with and without a history of stroke to examine a fundamental assumption in rehabilitation: that gait kinematics measured in the clinic reflect gait kinematics observed in real-world environments. This foundational principle is consequential in rehabilitation medicine. Clinicians observe and assess gait in the clinical setting and use this information to make decisions that have substantial consequences for a person’s daily life. Information gathered during a clinical gait assessment may influence whether an individual is prescribed a cane or a walker upon discharge from rehabilitation, whether they can safely return home or need to remain in rehabilitative care, and what activities they are encouraged or permitted to do independently. Because these decisions affect mobility, independence, safety, and participation in everyday life, it is essential that clinical gait assessments accurately represent how a person moves outside the clinic. Thus, we are specifically investigating this consequential assumption.

In this study, we aimed to determine 1) the relationship between gait measured in the clinic and gait measured in the home and 2) whether there is a statistical difference between the two environments for people with and without a history of stroke. This information is crucial for ensuring that clinical gait assessments meaningfully inform post-stroke rehabilitation planning affecting one’s safety, participation, and independence. We hypothesized that for individuals with and without a history of stroke, gait kinematics measured in the clinic would show weak correlations with and be statistically different from gait kinematics measured in the home due to environmental and contextual differences. We hypothesized that people would exhibit faster gait speed and lower step-length and step-time asymmetry, in the clinic than at home because clinical assessments occur under standardized, supportive conditions—firm, level flooring; consistent lighting; and clinician observation. In contrast, home environments may include variable surfaces (e.g., carpet, hardwood, linoleum) and lighting, environmental obstacles (e.g., furniture and pets), and less direct supervision, which could contribute to slower and more asymmetric gait. This work offers new insights into post-stroke mobility by evaluating whether clinical gait assessments reflect real-word walking behavior which may ultimately improve clinical decision-making regarding patients’ safety, participation, and independence.

## Methods

### Participants

Eighteen adults with a history of stroke  ≥  six months prior (Group A: 11 male; 66.72 ± 14.72 years old) and twenty-five adults without a history of stroke (Group B: 6 male; 72.4 ± 8.59 years old) are included in this study. All participants met the inclusion/exclusion criteria (S1 Table). The study protocol was approved by the Western University of Health Sciences Institutional Review Board and was conducted in compliance with the Declaration of Helsinki. All participants provided written informed consent prior to beginning the study.

### Experimental design

Each participant engaged in one in-clinic data collection session and one at-home data collection session. During the in-clinic data collection, participants underwent a standardized neurological clinical examination and completed a video-recorded walking assessment. The in-home data collection consisted of video-recorded walking using the same recording device employed during the in-clinic data collection session. Participants who typically wear ankle-foot orthoses and/or rely on an assistive device for ambulation used these supports throughout this study. Fig 1 provides an overview of data collection and analysis.

**Fig 1:**
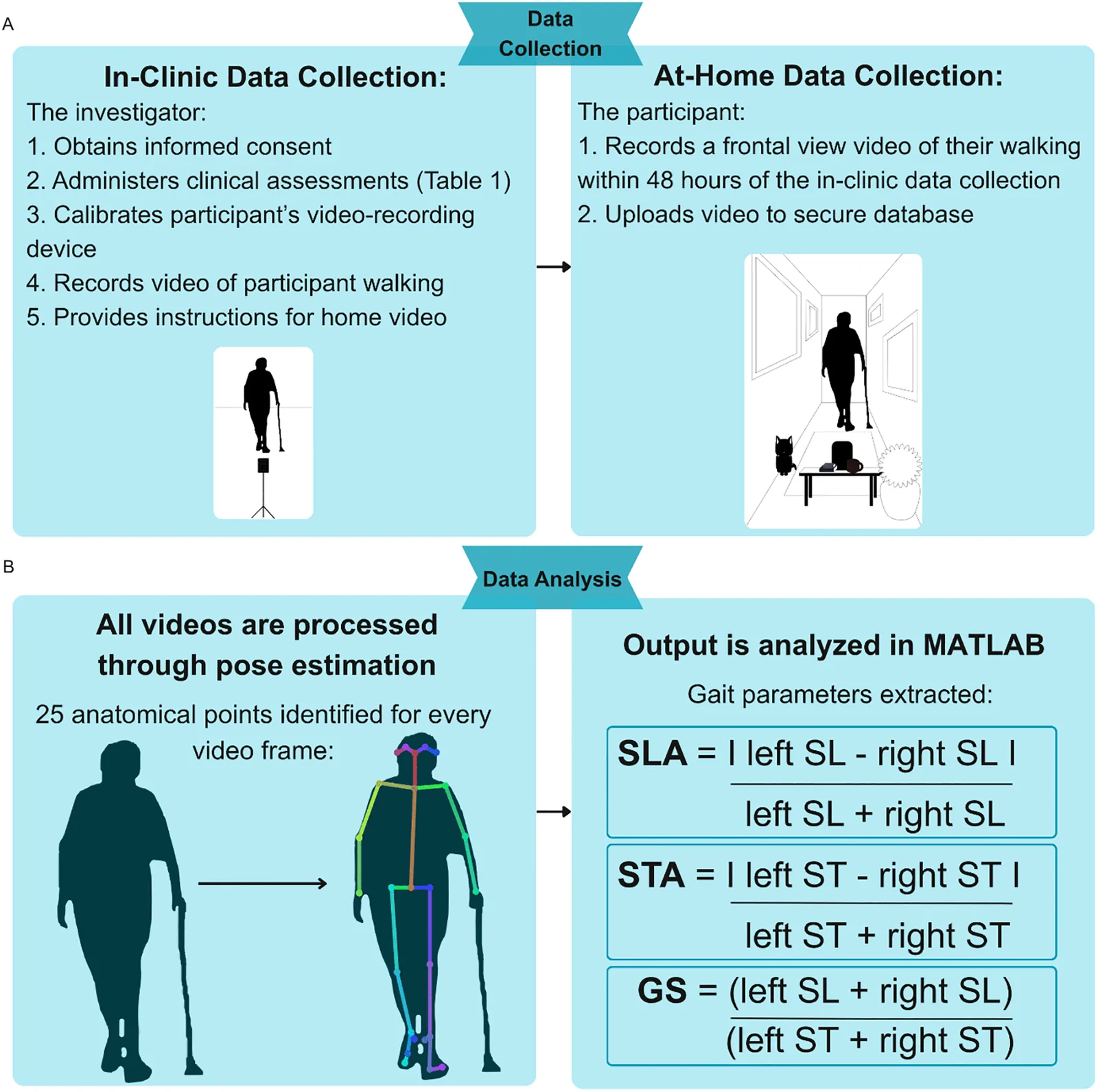
Data collection and analysis processes for gait parameters. (A) Depiction of the data collection process. (B) Simplified explanation of the data analysis process. ST:step time, duration of time in seconds between contralateral heel strikes; SL:step length, distance traversed by trunk between contralateral heel strikes measured in meters; SLA:step length asymmetry; STA:step time asymmetry; GS:gait speed, meters/second.

### Clinical assessments

The standardized neurological examination completed during the in-person data collection session assessed a spectrum of domains (Table 1). One assessment pertains to stroke incidence and was therefore administered only to Group A. Clinical Assessment data were entered directly into a secure electronic database: WesternU Qualtrics (www.qualtrics.com).

**Table 1:** Clinical assessment tools.

Clinical Assessment	Domain	Administered to
Comfortable walking speed	Physical	All participants
Patient Health Questionnaire 9 (PHQ-9) [18,19]	Psychosocial; emotional	All participants
Berg Balance Scale (BBS) [20,21]	Physical	All participants
Fugl-Meyer Assessment Lower Extremity (LE-FM) [22]	Physical	Group A
Mini-Mental State Examination (MMSE) [23,24]	Cognitive	All participants

### Video calibration procedures

A participant’s personal smartphone or tablet was used to record video data for this study. The same video-recording device was used for a given participant’s video calibration, in-clinic video recording, and in-home video recording. If a participant did not have a personal smartphone or tablet device with video-recording capacity, a loaner iPad was used. The autofocus system of the video-recording device was disabled before beginning video recording procedures.

Video recordings were calibrated using a two-step procedure to relate pixel height of a person to the distance from the camera allowing conversion of pixel-based measurements in the recordings to metric units for downstream kinematic analyses. The two-step calibration, depicted in S2 Fig, consisted of a camera calibration and a walking calibration for each participant. The camera calibration only occurred once, as the same camera was used in both settings. The walking calibration occurred for each participant in each setting. Initially, the camera focal length f (in pixels) was estimated by recording a planar object with known physical dimensions s positioned at a known distance $d_{\text{Ref}}$ from the camera and measuring its corresponding height in pixels $s_{\text{Ref}}$ in the image. The focal length was then computed as $f=(s_{\text{Ref}}\times d_{\text{Ref}})/s$, and this process was repeated across multiple known object distances; the mean value of f was used for subsequent analyses. Specifically, we used a piece of United States letter-sized paper taped to the wall as the planar object of known physical dimensions and recorded at distances of two, four, and six meters from the object by moving a tripod on which the camera was mounted.

For the walking calibration, an anthropometric measure with known physical height (the participant’s body height, which was measured in the clinic) was used as the reference object. Participants were instructed to stand still for five seconds at the beginning of each recording, ensuring the full reference object was visible. The pixel height of this reference ($s_{\text{Ref}}$) was extracted, and the initial camera-to-participant distance $d_{\text{Ref}}$ was estimated using $d_{\text{Ref}}=(s\times f)/s_{\text{Ref}}$, where s is the known physical height of the anthropometric measure. Initial camera-to-participant distance was then used to calculate step lengths in gait analysis.

### In-clinic video-recording procedures

A stationary smartphone or tablet was used to record digital video of each participant walking in the clinic at a frequency of thirty Hz using the outward-facing camera. The video-recording device was positioned on a tripod nine meters in front of the participant. The participant was asked to walk toward and away from the video-recording device repeatedly across a length of six meters for two minutes.

### At-home video-recording procedures

Within forty-eight hours after in-clinic data collection, participants recorded and submitted a video of themselves walking in their home environment. The same video-recording device used in the clinic was used to capture their walking at home. The participants received verbal and written instructions about how to complete home video recordings. Participants were instructed to 1) use the outward-facing camera, 2) record five seconds of standing still before beginning to walk, 3) ensure the camera would remain stationary while recording their walking, 4) walk toward and away from the camera for two minutes, and 5) use the provided quick response (QR) code to upload videos to a secure one-way SharePoint folder on the University’s server. The written instructions are included as S3 Fig. To capture the most ecologically valid home walking data, there were no investigators present during the home-video recordings, and participants were not told where to walk within their home environment. During home-video recording, investigators were able to be reached by phone if necessary. No participants required assistance during at-home data collection.

### Data processing and analysis

All two-dimensional frontal-plane video files, from both clinic and home settings, underwent two phases of processing. 1) The video files were run through freely-available pose estimation software: the OpenPose BODY_25 workflow [15] to automatically identify and mark twenty-five specific key anatomical points on the participant in every frame of every video creating JSON output files [13]. 2) The JSON output files were refined and data were extracted using MATLAB [25]. The custom MATLAB [25] program included a calibration component to promote consistency with data extraction from the video files despite use of different video-recording devices between participants.

In MATLAB [25], all data were run through code that checked the JSON files for accuracy, then through code to extract gait parameters [13,25,26]. For each video, up to four walking passes in which the participant walked toward the camera were analyzed individually. Gait parameters extracted from these passes were then averaged within each participant; therefore, all participants contributed a single mean value for each gait parameter to the statistical analyses. The primary gait parameters of interest were average step time (right and left), average step length (right and left), step length asymmetry (SLA), step time asymmetry (STA), and gait speed (GS). Equations are provided in Fig 1B.

### Statistical analysis

The primary purpose of this study was to understand the relationship between gait kinematics measured in the clinical setting and in the home setting among community-dwelling adults with and without a history of stroke. To address this aim, correlation coefficients were calculated for each gait parameter of interest (SLA, STA, and GS) to quantify the strength and direction of the association between in-clinic and in-home measurements. The interpretive scale used for correlation coefficients follows the general guidelines proposed by Schober and colleagues in their 2018 study: Correlation coefficient values between 0 and 0.1 represent a negligible relationship, between 0.1 and 0.4 indicate a weak relationship, between 0.4 and 0.7 suggest a moderate relationship, between 0.7 and 0.9 reflect a strong relationship, and coefficient values between 0.9 and 1.0 indicate a very strong relationship [27].

We conducted secondary analyses to complement the primary correlations. First, we examined whether gait parameters measured in the clinic and at home differed significantly within each group. This directly assessed whether gait patterns showed equivalence across environments beyond mere association. Second, we evaluated whether gait parameters differed significantly between groups within each setting. This explored whether gait dysfunctions that result from stroke appear differently across environments and whether clinical or real-world measures better captured group differences. Finally, as a sensitivity analysis, we fit generalized estimating equation (GEE) models to directly test the main effects of group and environment and the group × environment interaction while accounting for within-participant correlation across settings.

Data were screened before analyses to check key assumptions. Normality was assessed via Shapiro-Wilk tests (p > 0.05) and linearity was confirmed via scatterplots for correlations. Homogeneity of variance was examined with Levene’s test and independence of observations was ensured across all participants. Ninety-five percent confidence intervals (CIs) are reported for correlation coefficients (computed using the Fisher r-to-z transformation) and for the Hodges-Lehmann estimates of paired differences from Wilcoxon signed-rank tests to aid interpretation of effect magnitude and precision. Statistical significance was determined at α = 0.05.

## Results

### Demographics

A total of 53 individuals were screened and allocated into Group A, participants with a history of stroke, or Group B, participants without a history of stroke (S4 Fig). Two participants in Group A and one participant in Group B did not complete the study. Regarding participants who completed the study, data from three individuals in Group A and four individuals in Group B were excluded from analysis due to insufficient data quality. Issues included pixelated or blurry videos, failure to keep the recording device stationary, or inaccurate identification of key landmarks during pose estimation. Ultimately, the data from 43 participants—18 in Group A and 25 in Group B—were included in data analysis and are presented in this results section. The demographic information for these participants is shown in S5 Table along with the means and standard deviations for clinical assessment data. The extracted gait parameters, which include absolute metrics of step length, step time, and gait speed as well as differential metrics of step length asymmetry and step time asymmetry, are displayed in Table 2. Means of step length and step time are provided as *shorter* and *longer* rather than *left* and *right* as the shorter step was not consistently associated with the same limb across participants. This presentation of the data was used to characterize the magnitude of between-step differences independent of laterality.

**Table 2:** Absolute values of mean gait parameters for groups in-clinic and at-home.

	Group A In-Clinic Mean(SD)	Group A In-Home Mean(SD)	Group B In-Clinic Mean(SD)	Group B In-Home Mean(SD)
**Shorter Step Length, m**	0.44 (0.20)	0.42 (0.25)	0.53 (0.13)	0.52 (0.20)
**Longer Step Length, m**	0.50 (0.19)	0.53 (0.27)	0.57 (0.14)	0.56 (0.22)
**Shorter Step Time, sec**	0.69 (0.19)	0.66 (0.23)	0.55 (0.07)	0.53 (0.05)
**Longer Step Time, sec**	0.90 (0.57)	0.87 (0.52)	0.58 (0.10)	0.57 (0.07)
**Step Length Asymmetry**	0.12 (0.11)	0.15 (0.11)	0.07 (0.04)	0.07 (0.04)
**Step Time Asymmetry**	0.10 (0.14)	0.11 (0.15)	0.03 (0.02)	0.04 (0.02)
**Gait Speed, m/s**	0.70 (0.38)	0.73 (0.47)	1.00 (0.28)	0.99 (0.38)

Group A:history of stroke, Group B:no history of stroke, SD:standard deviation, m:meters, sec:seconds, m/s:meters per second.

### Statistical assumptions

Data normality was assessed for each planned analysis using the Shapiro-Wilk test. The correlation data violated normality assumptions (p < α), so nonparametric Spearman’s correlation was used. Scatterplots confirmed linear relationships for all variable pairs. For the paired-samples analysis, data did not meet normality criteria (p < α) resulting in use of the non-parametric Wilcoxon signed-rank Test. For the independent-samples analysis, data violated normality assumptions (p < α) and Levene’s test (p < α) indicating unequal variances between participants with and without a history of stroke, justifying use of the nonparametric Mann-Whitney U test.

### Primary analysis: Are gait parameters measured in the clinic correlated with gait parameters measured at home?

To address our primary aim, we examined the relationship between in-clinic and at-home gait parameters using within-group Spearman correlations (𝜌)*.* Overall, our findings suggest there is a statistically significant relationship between step time asymmetry and gait speed in-clinic and at-home in both groups (Table 3, Fig 2). In participants with a history of stroke (Group A), step length asymmetry and step time asymmetry were moderately correlated between the two environments (SLA: 𝜌 = 0.455, p = 0.058; STA: 𝜌 = 0.519, p = 0.027) while gait speed had a strong correlation (𝜌 = 0.752, p=<0.001). In participants without a history of stroke (Group B), there was a weak correlation between step length asymmetry in the clinic and at home (𝜌 = 0.116, p = 0.580) and a moderate correlation between step time asymmetry and gait speed in the clinic and at home (STA: 𝜌 = 0.406, p = 0.044; GS: 𝜌 = 0.541, p = 0.005).

**Table 3:** Spearman correlation results for within group comparison of gait parameters in-clinic and at-home.

	Group A			Group B		
	𝝆	CI	p-value	𝝆	CI	p-value
**SLA**	0.455	−0.014 to 0.760	0.058	0.116	−0.287 to 0.484	0.580
**STA**	0.519	0.067 to 0.794	0.027*	0.406	0.110 to 0.692	0.044*
**GS**	0.752	0.429 to 0.905	<0.001*	0.541	0.178 to 0.178	0.005*

Group A:participants with a history of stroke, Group B:participants without a history of stroke, p:Spearman’s rho, CI:95% Confidence Interval, SLA:step length asymmetry, STA:step time asymmetry, GS:gait speed, *p < 0.05). Mean gait outcomes are provided in Tables 1 and 4.

**Fig 2:**
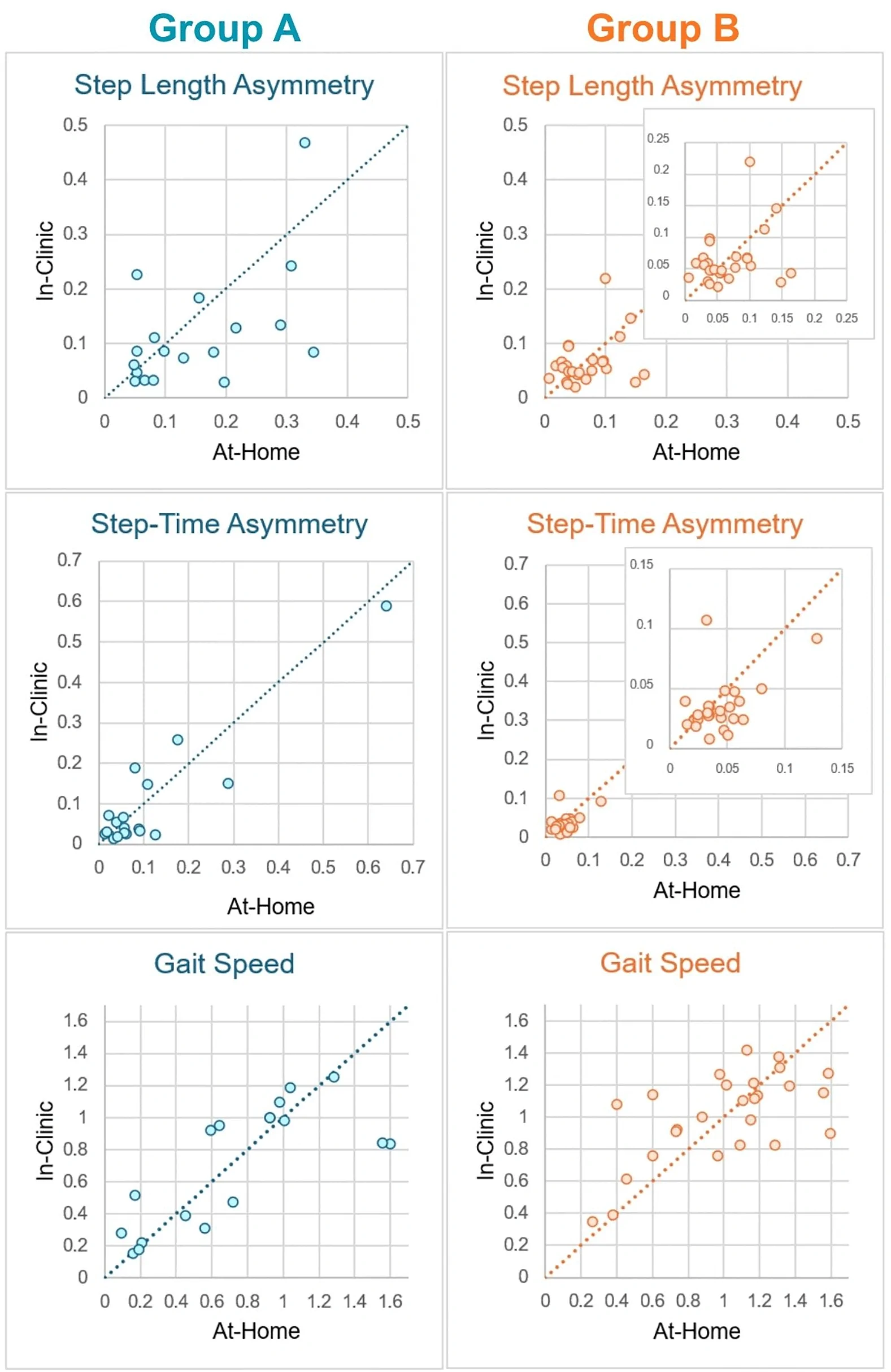
Scatterplots comparing in-clinic and at-home gait parameters for each group. Group A: participants with a history of stroke; Group B: participants without history of stroke; m/s: meters per second.

### Secondary and exploratory analyses

#### I. Are gait parameters measured in clinic statistically different from gait parameters measured at home?

To provide deeper insight beyond correlation, we conducted a related samples Wilcoxon signed-rank test within groups to determine whether gait parameters differed statistically between clinic and home settings (Table 4). No statistically significant differences were observed between in-clinic and at-home gait parameters for participants with a history of stroke. Step length asymmetry and gait speed did not exhibit statistically significant differences for participants without a history of stroke; however, step time asymmetry was statistically different between the clinic and home for participants without a history of stroke.

**Table 4:** Descriptive statistics and related samples Wilcoxon signed-rank test results for in-clinic and at-home gait parameters within groups.

Group A						
	In-clinic mean (SD)	At-home mean (SD)	𝒁_calc_	df	CI	p-value
**SLA**	0.12 (0.11)	0.15 (0.11)	1.46	17	−0.01 to 0.08	0.14
**STA**	0.10 (0.14)	0.11 (0.15)	0.98	17	−0.02 to 0.04	0.33
**GS**	0.70 (0.38)	0.73 (0.47)	−0.28	17	−0.14 to 0.14	0.78
**Group B**						
	**In-clinic** mean (SD)	**At-home** mean (SD)	**𝒁**_**calc**_	**df**	**CI**	**p-value**
**SLA**	0.07 (0.04)	0.07 (0.04)	0.79	24	−0.01 to 0.02	0.43
**STA**	0.03 (0.02)	0.04 (0.02)	2.68	24	0.00 to 0.02	0.01*
**GS**	1.00 (0.28)	0.99 (0.38)	−0.23	24	−0.13 to 0.11	0.82

Group A:participants with a history of stroke, Group B:participants without a history of stroke, SD:standard deviation, 𝑍_calc_:test statistic, df:degrees of freedom, CI:95% confidence interval, SLA:step length asymmetry, STA:step time asymmetry, GS:gait speed, *p < 0.05.

#### II. Are gait parameters statistically different between people with and without a history of stroke in the clinic or the home?

Given the novel application of this technology within the home environment, it was important to determine whether the measurements could distinguish between groups known to differ in gait patterns. Consistent with our a priori hypotheses, significant differences were observed between participants with and without a history of stroke for all gait parameters in both the clinic and home environments. These findings provide additional evidence supporting the validity of measurements collected in participants’ homes. Additionally, participants with a history of stroke exhibited slower and more asymmetric gait overall as compared to participants without a history of stroke across settings (Table 5).

**Table 5:** Results of Mann-Whitney U test comparing gait parameters between groups.

	Median		U	𝒁_calc_	n	p-value
	Group A	Group B				
**Clinic SLA**	0.09	0.05	307	2.02	43	0.04*
**Clinic STA**	0.04	0.03	305	1.97	43	0.05*
**Clinic GS**	0.84	1.08	123	−2.51	43	0.01*
**Home SLA**	0.11	0.05	341	2.86	43	<0.01*
**Home STA**	0.06	0.03	317	2.26	43	0.02*
**Home GS**	0.68	1.09	143	−2.02	43	0.04*

U:Mann-Whitney U test statistic, 𝑍_calc_:standardized test statistic, n:sample size, SLA:step length asymmetry, STA:step time asymmetry, GS:gait speed, *p < 0.05.

A sensitivity GEE analysis yielded results consistent with the primary nonparametric analyses: a significant main effect of group was observed for all gait parameters (all p < 0.05) and no significant main effects of environment (SLA p = 0.137; STA p = 0.172; GS p = 0.817) or group × environment interactions (SLA p = 0.278; STA p = 0.933; GS p = 0.688) were identified. These findings did not alter the overall interpretation of the results.

## Discussion

### Summary of key findings

The primary purpose of this study was to determine the relationship between gait kinematics measured in the clinical setting and in the home setting in community-dwelling adults with and without a history of stroke. Using two-dimensional frontal-plane video recordings, we found weak-to-strong correlations between gait parameters measured in the clinical setting and in the home setting in both groups. We also found that most gait parameters did not differ significantly between in-clinic and at-home assessments. These findings indicate that clinic-based gait assessments may provide useful insight into gait exhibited in daily life; however, variability in the strength of these relationships highlights that clinic-based measures do not fully reflect real-world gait for all individuals.

### Substantiation of at-home measurement of gait kinematics

Clinicians and researchers have long worked to develop and refine measurement tools capable of capturing movement as it occurs in the real world with particular focus on the unique challenges and opportunities presented by the community and home environments. For example, the Kinect has been used to measure gait speed and various gait parameters in clinical and laboratory settings with the intention of ultimately using this technology to measure gait in the real world in elderly adults [28], adults with Parkinson’s disease [29], and adults with a history of stroke [30]. Wearable sensors have also been used to quantify functional activities such as number of steps and activities of daily living in the home for people with Parkinson’s disease, a history of stroke, multiple sclerosis, and frailty in the real world [12]. While data gathered using each of these methods is informative, these tools have notable limitations, including high cost, cumbersome set-up, and challenges with wearability. Further, these methods often capture only a limited aspect of actual in-home mobility.

This study represents a critical progression from previous work as it directly compares measures of gait kinematics in participants’ homes with measures of gait kinematics in the clinical setting. This allowed us to assess the fundamental assumption that gait measured in the clinic reflects real-world gait. The processes employed in this study involve minimal barriers, indicating strong feasibility for implementation by others. Most participants used their personal smartphone or tablet for recording videos, which reduced major barriers by leveraging devices they already owned and eliminating additional costs. Set-up was simple as the participant only needed to position the device to be stationary while recording themselves walking. This method did not require participants to wear additional equipment or specific clothing. Moreover, most people were familiar with the basic video function on their devices, and those who were not were able to quickly learn.

All video data reported in this study were processed using freely available pose estimation software [15] and a custom MATLAB [25] program. A total of 50 home videos were submitted, and 43 resulted in usable data (86%). The primary reasons for exclusion were poor video quality with visible pixelation and occasional failures of the pose estimation algorithm to reliably distinguish the participant from background objects and people. These findings demonstrate the feasibility of obtaining useful kinematic data with limited resources and the practicality of capturing real-world gait. The ability to directly measure gait in an individual’s natural environment opens new opportunities to evaluate mobility with greater ecological validity.

### Interpretation of within group differences

The results of this study indicate that gait parameters measured in the clinic and in the home are correlated to varying degrees (weak-to-strong), and that most parameters do not differ significantly between settings. These findings contrast with our initial hypotheses, which proposed that these measures would not be correlated and would differ significantly due to environmental factors. One potential explanation for such differences is reinvestment theory, which proposes that directing conscious attention toward movement may alter it [31]. Because clinical assessments are conducted in structured environments and involve direct observation by clinicians and researchers, gait measured in the clinic may reflect greater conscious control of movement than gait performed in more naturalistic settings. Although reinvestment was not assessed directly, this framework provides one possible explanation for why clinic- and home-based gait assessments may differ for some individuals. However, the generally similar gait parameters observed across settings suggest that any such effects were limited for the gait measures examined in this study.

Prior work by Waddell and colleagues demonstrated that improvements in upper limb use in clinical settings did not inevitably translate to increased upper limb use in the real-world, indicating a disconnect between clinical and real-world settings [32]. Benson and colleagues also noted that relatively few studies have incorporated movement data collected outside laboratory environments [33]. Additionally, Rispens and colleagues reported significant differences between gait data collected via accelerometry in clinical and home settings [34].

Discrepancies between our findings and prior studies create an opportunity for further discussion. These results suggest that gait measured in the clinical setting may provide useful insight into gait in the home environment. However, variability in the strength of associations between the clinic and home environments highlight that clinic-based assessments do not fully reflect movement in the real-world gait for all individuals. Home-based kinematic gait measurement therefore remains a valuable tool for capturing ecologically valid gait. The ability to measure gait kinematics in natural environments can support more informed clinical decision-making and facilitate personalized interventions that promote safety, independence, function, and participation.

This variability may help explain why, despite prior reports of differences between clinical and home settings, we observed relatively consistent gait parameters across environments. Hug and colleagues reported strong associations between clinical walking assessments and daily distance walked in participants with a history of stroke or spinal cord injury; however, they also found substantial unexplained variability among participants with “moderate to good walking ability.” These findings indicate clinical walking assessments alone may not be sufficient to fully distinguish real-world walking [35]. Similarly, Bhatnagar and colleagues found that although accelerometer-measured paretic upper extremity use at home was significantly correlated with clinically assessed impairment-level in participants with a history of stroke, individuals with higher functional levels did not consistently demonstrate greater use of their paretic upper extremity at home. Taken together, these findings suggest that while clinical gait assessments provide valuable insight, it is beneficial to supplement real-world measurements to more fully characterize authentic walking and to inform individualized rehabilitation strategies.

### Interpretation of differences between groups

The differences in gait kinematics between people with and without a history of stroke have been extensively described [36,37]. The results of our between-group comparison of gait parameters demonstrated that the methodology used in this study detected expected differences in gait kinematics as there were statistically significant differences in all gait parameters between groups in both settings. Although not a primary objective of the study, the observed between-group differences were consistent with previously reported gait differences between individuals with and without a history of stroke. This finding provides additional context regarding the clinical meaningfulness of the measurements derived from the pose estimation approach.

### Implications

Collectively, these results indicate that measuring gait kinematics in the clinical setting can provide valuable insight into real-world gait. However, not all individuals demonstrated strong correlations between clinical and home settings, highlighting important variability in how well clinic-based measures reflect real-world movement. Home-based kinematic gait measurement therefore remains a valuable tool for capturing ecologically valid gait. The ability to measure gait kinematics in natural environments can support more informed clinical decision-making and facilitate personalized interventions that promote safety, independence, function, and participation. This study contributes to this area by incorporating real-world data collection and using validated, freely available technology to assess gait kinematics in community-dwelling adults with and without a history of stroke [13]. These findings establish important groundwork for future research and for the development of alternative approaches to measuring gait kinematics in real-world settings, ultimately supporting more individualized and ecologically valid physical therapy care.

### Limitations and future directions

Although this study provides significant quantitative results, limitations must be noted. First, videos used to extrapolate gait kinematics were from a single frontal plane camera. While sagittal plane videos are typically preferred in laboratory assessments, this is not feasible in the home environment due to space constraints. Instead, frontal plane recordings were compatible with these settings and provided ecologically valid data with acceptable accuracy [26]. This approach allowed for practical data collection while preserving measurement quality in real-world circumstances. Second, the participants within this study had limited diversity. A larger and more diverse sample of participants would enhance generalizability. Third, while we were able to gather data in the real world within participant’s own homes, they were aware video was being taken, which could inherently affect their gait. Keeping these limitations in mind, these findings open new avenues for research by demonstrating the feasibility of measuring gait kinematics in the home setting using accessible technology. This study informs future research that can investigate critical questions such as whether improvements in gait mechanics achieved in the clinic translate to the home environment, whether these changes persist over time, and whether gait quality or quantity is more important for functioning in the real world for individuals with a history of stroke. With validated home-based measurement tools, researchers can now pursue the answers to these essential questions.

## Conclusions

At-home measurement of gait kinematics is both feasible and informative providing useful insight into real-world gait for adults with or without a history of stroke. This study highlights the clinical and research potential of home-based gait assessment technologies to improve access to, accuracy of, and ecological validity in the evaluation of human gait. These findings also reveal that gait observed in the clinic provides insight into real-world gait.

## Supporting information

S1 Table Inclusion and exclusion criteria. *This criterion only applies to participants with a history of stroke.
(TIF)

S2 Fig Video-recording device calibration and walking calibration procedures schematic with formulas. (TIF)

S3 Fig Written instructions for participants regarding home video data collection. (TIF)

S4 Fig Modified CONSORT flow diagram. (TIF)

S5 Table Demographic and clinical assessment data. Group A:history of stroke, Group B:no history of stroke, SD:standard deviation, AFO(s):ankle-foot orthosis(ses), N/A:not applicable, m/s:meters per second, Table 1 conveys meaning of clinical assessment abbreviations.
(TIF)
