# Supplementary material for: In-clinic versus at-home gait kinematics in adults with and without stroke: Associations across environments
Source: PLoS One. 2026 Nov 11;21(11):e0361481. doi: 10.1371/journal.pone.0361481 (PMC13754162; doi:10.1371/journal.pone.0361481)
Supplement: Supplementary file 1 [file pone.0361481.s007.docx]

Reviewer's Responses to Questions

**Comments to the Author**

Reviewer #1:

*Summary*

The research here reports a null finding with respect to differences between clinic and at-home gait kinematics in people with and without stroke. This investigation of the effects of changing the data-gathering environment for gait parameters is novel, and provides results that strengthen clinic-based gait measurement as estimating ‘true’ gait properties.
 A significant part of the value of the current work is continuing to demonstrate to researchers involved with gait rehabilitation the power, efficiency, availability and low cost of Pose Estimation-based gait analysis. The paragraphs describing and supporting this method in the Introduction succinctly summarise supporting work published since the development of Pose Estimation.

Thank you for your positive comments. We appreciate your recognition of the novelty of this work and the potential value of pose estimation-based gait analysis for gait research and clinical applications. We are pleased that the rationale and significance of the study were clearly conveyed.

*Potential Influence of Reinvestment on Clinic-Based Gait Performance*

However, there is a body of work that could support the logic for investigating the effects of environment change on gait parameters, and that is the work on Reinvestment conducted by Rich Masters and colleagues (Masters, R., & Maxwell, J. (2008). The theory of reinvestment. International Review of Sport and Exercise Psychology, 1(2), 160-183. Young, W. R., Olonilua, M., Masters, R. S., Dimitriadis, S., & Mark Williams, A. (2016). Examining links between anxiety, reinvestment and walking when talking by older adults during adaptive gait. Experimental brain research, 234(1), 161-172). This work highlights the possibility that gait measurement undertaken in clinics is assessing a consciously-controlled form of the target.
 Movie directors, particularly those making Westerns, often struggled with actors whose walking gait became affected when shooting scenes. John Wayne was famously impatient with young actors who were unable to walk naturally on the movie set. Masters was one of the first researchers to call attention to the interfering effects of directing conscious attention to movement, terming the mechanism ‘reinvestment’.
 So reinvestment was a real prospect for confounding in-clinic gait assessment, with the outcome being that the measures did not reflect the participants ‘real’ gait.
 The results here indicate that reinvestment effects did not occur. The authors should add text taking credit for ruling out this possibility.

Thank you for this thoughtful suggestion. We agree that the reinvestment literature provides a relevant theoretical framework for considering potential differences between clinic- and home-based gait assessment. As the reviewer notes, characteristics of the clinical environment, including direct observation by clinicians and researchers, may increase conscious attention to movement and potentially influence gait performance.

We have incorporated discussion of this concept into the manuscript. Although reinvestment was not directly assessed, our findings suggest that clinic-based assessments provide meaningful information regarding real-world gait performance while not fully reflecting gait behavior within the home environment. We appreciate the reviewer highlighting this relevant theoretical framework.

***Interpretation of Within Group Differences***

*“The results of this study indicate that gait parameters measured in the clinic and in the home are correlated to varying degrees (weak-to-strong), and that most parameters do not differ significantly between settings. These findings contrast with our initial hypotheses, which proposed that these measures would not be correlated and would differ significantly due to environmental factors. One potential explanation for such differences is reinvestment theory, which proposes that directing conscious attention toward movement may alter it.(31) Because clinical assessments are conducted in structured environments and involve direct observation by clinicians and researchers, gait measured in the clinic may reflect greater conscious control of movement than gait performed in more naturalistic settings. Although reinvestment was not assessed directly, this framework provides one possible explanation for why clinic- and home-based gait assessments may differ for some individuals. However, the generally similar gait parameters observed across settings suggest that any such effects were limited for the gait measures examined in this study.”*

Reviewer #2:

*CONCEPTUAL FRAMING*

Major comments, 1. The motivation for this work needs to be more clear. The second paragraph of the introduction discusses the discrepancy between capacity (i.e, if they can) and performance (i.e., if they do), but the majority of metrics in this work are at the impairment level. The connection between this discrepancy and measures of impairment needs to be clear if this is what motivates the work.

Major comments 12. In the discussion, the author circle back to this distinction between capacity and performance. I remain unclear of how this work connects to that previous work as the authors are measuring an entirely different ICF domain (impairment) and they are measuring the same domain just in a different environment as opposed to 2 different domains.

Thank you for the feedback. We have clarified the study motivation in the Introduction and revised the manuscript as a whole to avoid conflating the capacity–performance framework with the impairment-level gait outcomes examined in this study. Specifically, we removed references to “performance” and refocused the rationale on whether gait characteristics measured in the standardized clinical environment differ from those observed in the home environment. We also revised terminology throughout the manuscript to use “gait dysfunction” and “gait impairment” consistently and to distinguish environmental differences in gait from broader constructs of capacity and performance.

***Introduction***

*“Post-stroke gait dysfunctions—including slowed gait speed and gait asymmetries— have been shown to worsen over time following discharge from rehabilitation, highlighting the importance of follow-up and longitudinal assessment of mobility within a person’s natural environment.(6) Furthermore, capturing gait metrics that reflect how people walk in the real world is essential for developing interventions that truly restore mobility after stroke. Standard clinical assessments, typically conducted in controlled settings, are commonly used to estimate walking ability.(10) These tests measure how an individual performs under ideal conditions and require administration by a trained clinician. For example, the 6-Minute Walk Test and the 10-Meter Walk Test measure what individuals can do in the clinic rather than what they actually do in their daily environment.(5,11) Consequently, a person may demonstrate the capability to ambulate in the community during testing yet remain inactive once home. More detailed gait information at the impairment level (e.g. joint kinematics, spatiotemporal gait parameters) can be obtained using three-dimensional motion capture systems, but these approaches are expensive and limited to specialized laboratory environments.(12) Thus, there is a critical need for assessment tools that are low-cost, accessible, and capable of objectively capturing how individuals move in real-world settings.”*

*And*

*“We leveraged freely available pose estimation(15) technology to capture real-world gait metrics in people with and without a history of stroke to examine a fundamental assumption in rehabilitation: that gait kinematics measured in the clinic reflect gait kinematics observed in real-world environments. This foundational principle is consequential in rehabilitation medicine. Clinicians observe and assess gait in the clinical setting and use this information to make decisions that have substantial consequences for a person’s daily life. Information gathered during a clinical gait assessment may influence whether an individual is prescribed a cane or a walker upon discharge from rehabilitation, whether they can safely return home or need to remain in rehabilitative care, and what activities they are encouraged or permitted to do independently. Because these decisions affect mobility, independence, safety, and participation in everyday life, it is essential that clinical gait assessments accurately represent how a person moves outside the clinic. Thus, we are specifically investigating this consequential assumption.*

*In this study, we aimed to determine 1) the relationship between gait measured in the clinic and gait measured in the home and 2) whether there is a statistical difference between the two environments for people with and without a history of stroke. This information is crucial for ensuring that clinical gait assessments meaningfully inform post-stroke rehabilitation planning affecting one’s safety, participation, and independence. We hypothesized that for individuals with and without a history of stroke, gait kinematics measured in the clinic would show weak correlations with and be statistically different from gait kinematics measured in the home due to environmental and contextual differences. We hypothesized that people would exhibit faster gait speed and lower step-length and step-time asymmetry, in the clinic than at home because clinical assessments occur under standardized, supportive conditions—firm, level flooring; consistent lighting; and clinician observation. In contrast, home environments may include variable surfaces (e.g., carpet, hardwood, linoleum) and lighting, environmental obstacles (e.g., furniture and pets), and less direct supervision, which could contribute to slower and more asymmetric gait. This work offers new insights into post-stroke mobility by evaluating whether clinical gait assessments reflect real-word walking behavior which may ultimately improve clinical decision-making regarding patients’ safety, participation, and independence.”*

Major comments, 2. Justification for the hypotheses are needed. Why do the authors think how someone walks will be different just because they are at home vs in the lab. The conditions at home do not necessarily have to be different. For example, the floor in a lab/clinic is not very different than tile or hardwood floors that are often in homes. Additionally, if gait parameters are different is this necessarily bad? The introduction appears to couch differences in gait measurements in the lab vs in the community as a limitation/weakness to current measurement (paragraph 2 of the intro), but humans modify their gait in response to their environment regularly. I am unclear what the expected result would be given that there is no information about how similar/dissimilar the home environment is compared to the lab/clinic.

Thank you for this thoughtful comment. We revised the Introduction to provide a clearer rationale for our hypotheses and to clarify our interpretation of potential differences between clinical and home gait. Specifically, we now describe the clinic as a relatively standardized setting and the home as a setting that may involve variable contextual features, including lighting, flooring, space constraints, and obstacles (e.g., furniture and pets). We acknowledge that some home surfaces may be comparable to clinic flooring and that home environments vary substantially across individuals.

We also revised the manuscript to clarify that differences in gait characteristics across settings are not inherently maladaptive or indicative of poorer gait. Rather, individuals appropriately modify gait in response to environmental and contextual demands. The purpose of this study was therefore not to characterize home gait as “worse” than clinic gait, but to determine whether gait kinematics measured during a standardized clinical assessment correspond to those observed in an individual’s home environment. We added this rationale and the basis for our hypotheses to the Introduction. Historically, gait assessment has been conducted primarily in clinical settings, and clinical observations are often used to guide treatment decisions. However, demonstrating consistent walking patterns during a clinical assessment does not necessarily indicate that an individual will demonstrate the same walking patterns during daily activities. This potential discrepancy underscores the value of evaluating gait in real-world environments and highlights the need for clinicians to consider the extent to which clinic-based assessments reflect everyday mobility.

***Introduction***

*“We leveraged freely available pose estimation(15) technology to capture real-world gait metrics in people with and without a history of stroke to examine a fundamental assumption in rehabilitation: that gait kinematics measured in the clinic reflect gait kinematics observed in real-world environments. This foundational principle is consequential in rehabilitation medicine. Clinicians observe and assess gait in the clinical setting and use this information to make decisions that have substantial consequences for a person’s daily life. Information gathered during a clinical gait assessment may influence whether an individual is prescribed a cane or a walker upon discharge from rehabilitation, whether they can safely return home or need to remain in rehabilitative care, and what activities they are encouraged or permitted to do independently. Because these decisions affect mobility, independence, safety, and participation in everyday life, it is essential that clinical gait assessments accurately represent how a person moves outside the clinic. Thus, we are specifically investigating this consequential assumption.*

*In this study, we aimed to determine 1) the relationship between gait measured in the clinic and gait measured in the home and 2) whether there is a statistical difference between the two environments for people with and without a history of stroke. This information is crucial for ensuring that clinical gait assessments meaningfully inform post-stroke rehabilitation planning affecting one’s safety, participation, and independence. We hypothesized that for individuals with and without a history of stroke, gait kinematics measured in the clinic would show weak correlations with and be statistically different from gait kinematics measured in the home due to environmental and contextual differences. We hypothesized that people would exhibit faster gait speed and lower step-length and step-time asymmetry, in the clinic than at home because clinical assessments occur under standardized, supportive conditions—firm, level flooring; consistent lighting; and clinician observation. In contrast, home environments may include variable surfaces (e.g., carpet, hardwood, linoleum) and lighting, environmental obstacles (e.g., furniture and pets), and less direct supervision, which could contribute to slower and more asymmetric gait. This work offers new insights into post-stroke mobility by evaluating whether clinical gait assessments reflect real-word walking behavior which may ultimately improve clinical decision-making regarding patients’ safety, participation, and independence.”*

Major comments, 10. The summary of the conclusion at the beginning of the discussion is significantly oversimplified. Correlations range from weak to strong, so I am unclear how the findings support that “gait measured in the clinic provides meaningful insights into gait performance in daily life.”

We agree that our original summary statement oversimplified the findings, thank you for bringing this to our attention. While several gait measures demonstrated moderate to strong correlations between the clinic and home environments, others demonstrated only weak associations. Therefore, our findings suggest that clinic-based gait assessments may provide meaningful insight into some aspects of gait in daily life, but they do not fully capture gait behavior in the home environment. We have revised the Discussion to more accurately reflect the range of observed correlations and the implications of these findings.

***Discussion***

*“The primary purpose of this study was to determine the relationship between gait kinematics measured in the clinical setting and in the home setting in community-dwelling adults with and without a history of stroke. Using two-dimensional frontal-plane video recordings, we found weak-to-strong correlations between gait parameters measured in the clinical setting and in the home setting in both groups. We also found that most gait parameters did not differ significantly between in-clinic and at-home assessments. These findings indicate that clinic-based gait assessments may provide useful insight into gait exhibited in daily life; however, variability in the strength of these relationships highlights that clinic-based measures do not fully reflect real-world gait for all individuals.”*

*METHODS CLARIFICATION*

Major comments, 3. The Data Processing and Analysis section states that “up to four segments of the participant walking toward the camera” were analyzed. Is a segment a ‘walking pass?’ Does this mean that no data were analyzed when the participant was walking away from the camera? Conversely, “as many segments as possible” were analyzed if there were not 4 segments. Was there a minimum number of segments?

Thank you for requesting clarification. We have revised the manuscript to specifically state ‘walking pass’ rather than ‘segment’ for improved readability. We have also clarified that the algorithm analyzed gait only during walking passes in which participants walked toward the camera, with a minimum of one complete walking pass required for analysis. Please see the next section of red text below for these changes.

Major comments, 4. Each individual had multiple steps from which most of the gait metrics could be calculated. How was this handled in the analysis? Were all the steps averaged for each person to ensure independence of observations?.

Thank you for identifying this important point regarding the unit of analysis and independence of observations. We have revised the Data Processing and Analysis section to clarify that gait metrics were first calculated for each individual walking pass and then averaged within each participant. Thus, all participants contributed one value per gait parameter to the statistical analysis, ensuring that observations were independent.

***Data Processing and Analysis*:**

“*In MATLAB(25), all data were run through code that checked the JSON files for accuracy, then through code to extract gait parameters.(13,25,26) For each video, up to four walking passes in which the participant walked toward the camera were analyzed individually. Gait parameters extracted from these passes were then averaged within each participant; therefore, all participants contributed a single mean value for each gait parameter to the statistical analyses. The primary gait parameters of interest were average step time (right and left), average step length (right and left), step length asymmetry (SLA), step time asymmetry (STA), and gait speed (GS). Equations are provided in Figure 1B.*”

Minor comments, 5. Please include the assessments completed in the text rather than the supplemental materials.

We thank the reviewer for this suggestion. To improve readability and provide readers with immediate access to this information, we have moved the assessment descriptions from the supplemental materials into the main text as Table 1.

Minor comments, 6. Please provide a copy of the instructions provided to the participants as a supplemental.

We thank the reviewer for this helpful suggestion. To improve transparency and provide additional context regarding the study procedures, we have included a copy of the instructions provided to participants as Supplemental Material S3.

DATA REPORTING

Major comments, 5. The number of subjects does not match the number of exclusions shown in Figure 3 or described in the results.

We believe the reviewer is referring to Supplemental Material S4. Supplemental Material S4 and the corresponding error in the Results section have been revised to accurately reflect the study progression.

***Demographics***

*“A total of 53 individuals were screened and allocated into Group A, participants with a history of stroke, or Group B, participants without a history of stroke (Supplemental Material S4). Two participants in Group A and one participant in Group B did not complete the study. Regarding participants who completed the study, data from three individuals in Group A and four individuals in Group B were excluded from analysis due to insufficient data quality. Issues included pixelated or blurry videos, failure to keep the recording device stationary, or inaccurate identification of key landmarks during pose estimation. Ultimately, the data from 43 participants—18 in Group A and 25 in Group B—were included in data analysis and are presented in this results section. The demographic information for these participants is shown in Supplemental Material S5 along with the means and standard deviations for Clinical Assessment data.”*

Major comments, 7. I am unclear why the gait speeds presented in Table 3 are so different than the gait speeds in Table 2.

We appreciate the opportunity to clarify. Gait speed mean values are reported in Table 3 (now Table 4), whereas Table 2 (now Table 3) presents the Spearman correlation results. We have revised the manuscript to make this distinction clearer for readers. We also verified that the gait speed mean values reported in Table 1 (now Table 2) match Table 3 (now Table 4).

Minor comments, 3. I suggest stating what modified independent means in Table 2.

We appreciate the reviewer's suggestion. We believe the reviewer is referring to Supplemental Material S1. To improve clarity, we have added a brief definition of modified independence within Supplemental Material S1.

Minor comments, 4. I suggest adding the time component of 48 hours to Figure 1.

We thank the reviewer for this suggestion. Figure 1 has been revised to include the 48-hour time component.

Minor comments, 7. Significance is not indicated in Table 4 the same between groups.

Table 4 (now Table 5) includes p-values for all comparisons, and statistically significant values are indicated by an asterisk, as noted in the table footnote. We reviewed the table and footnote to ensure that this information is clearly presented.

OUTCOME CONSTRUCTION AND ASYMMETRY METRICS

Major comments, 6. Shorter and longer step time/length were used instead of left/right. Was the same leg always the shorter/longer? How was it handled if this wasn’t the case? I assume this could have occurred in the healthy group.

We have clarified how shorter and longer step metrics were defined. The limb associated with the shorter or longer step was not consistent across participants, including among healthy control participants. Therefore, left and right step measures were calculated for each participant and then ranked such that the smaller mean value was reported as the shorter step and the larger mean value was reported as the longer step. These labels do not represent a consistent anatomical side across participants and were used to characterize the magnitude of between-step differences independent of laterality. To avoid ambiguity, we have revised the Results section and Table 2 description accordingly.

***Demographics***

*“The extracted gait parameters, which include absolute metrics of step length, step time, and gait speed as well as differential metrics of step length asymmetry and step time asymmetry, are displayed in Table 2. Means of step length and step time are provided as shorter and longer rather than left and right as the shorter step was not consistently associated with the same limb across participants. This presentation of the data was used to characterize the magnitude of between-step differences independent of laterality.”*

STATISTICAL ANALYSES

Major comments, 8. Figure 4 shows that there appears to be a fairly significant outliner in the group of individuals with stroke on STA. A sensitivity analysis should be conducted to see how that individual is influencing the results.

Thank you for this observation. We reviewed the participant with the largest STA value in Group A and confirmed that the observed asymmetry was consistent with the participant's gait pattern and did not reflect a measurement or data-processing error. Given that substantial gait asymmetry is a clinically relevant characteristic in some individuals with stroke, we considered this observation to represent a valid point within the clinical spectrum rather than an irregular value.

To assess the influence of this participant on the results, we conducted a sensitivity analysis by repeating all Spearman correlation analyses after excluding the participant. Sensitivity analyses demonstrated that exclusion of the participant with the largest STA value resulted in only minor changes in correlation coefficients (mean absolute change in ρ=0.037), including the clinic-home STA relationship (ρ=0.477 vs. 0.439). The overall interpretation of the findings remained unchanged, supporting retention of this participant in the primary analysis.

Major comments, 9. I am unclear why the secondary analyses that were complete were done. Specifically, why were the gait metrics between groups compared? We know that individuals with stroke walk differently. Assessing the interaction between group and condition seems far more interesting and informative than the analyses done. I acknowledge that there is not a nonparametric test of a 2x 2 mixed ANOVA; however, there are other options (robust ANOVA, GEE, etc) that should be conducted.

*Why the secondary analyses?*

Thank you for this thoughtful comment. We agree that the primary aim of this study was to examine the relationship between clinic- and home-based gait measures and determine whether gait parameters differed between environments. However, because the application of pose estimation technology within the home environment is novel, it was also important to explore whether the resulting measurements could distinguish between groups known to differ in gait performance. Consistent with our a priori expectations, participants with a history of stroke demonstrated slower and more asymmetric gait than participants without a history of stroke. Although exploratory, these findings provide additional support that the measurements collected within participants' homes captured meaningful aspects of gait.

Because preliminary analyses demonstrated substantial violations of normality and homogeneity assumptions, the primary analyses used nonparametric statistical methods. We appreciate the reviewer's suggestion to explore a generalized estimating equation (GEE) approach. Although GEE is a parametric modeling framework, it permits direct evaluation of main effects and interaction effects and is generally considered more robust to assumption violations than traditional ANOVA-based approaches. Therefore, we conducted GEE analyses as a supplementary sensitivity analysis to determine whether the overall conclusions were consistent across analytic approaches.

The GEE analyses yielded conclusions consistent with those obtained from the primary nonparametric analyses. Specifically, significant main effects of group were observed, whereas no significant main effects of environment or group × environment interactions were identified. Thus, the interpretation of the findings remained unchanged. We have added clarification regarding the rationale for the exploratory analyses and included the GEE sensitivity analyses in the revised manuscript.

***Secondary and Exploratory Analyses: II.	Are gait parameters statistically different between people with and without a history of stroke in the clinic or the home?***

*“Given the novel application of this technology within the home environment, it was important to determine whether the measurements could distinguish between groups known to differ in gait patterns. Consistent with our a priori hypotheses, significant differences were observed between participants with and without a history of stroke for all gait parameters in both the clinic and home environments. These findings provide additional evidence supporting the validity of measurements collected in participants' homes. Additionally, participants with a history of stroke exhibited slower and more asymmetric gait overall as compared to participants without a history of stroke across settings (Table 5).”*

*and*

*“A sensitivity GEE analysis yielded results consistent with the primary nonparametric analyses: a significant main effect of group was observed for all gait parameters (all P<0.05) and no significant main effects of environment (SLA p=0.137; STA p=0.172; GS p=0.817) or group x environment interactions (SLA p=0.278; STA p=0.933; GS p=0.688) were identified. These findings did not alter the overall interpretation of the results.”*

Major comments, 13. In the discussion, the authors state that the between group difference support that PoseEstimation can detect expected differences in gait, but this is not a purpose of the work, so I again question the utility of this analysis.
 We agree that demonstrating between-group differences was not a primary aim of the study. However, because the application of this pose estimation approach within the home environment is novel, we considered it important to explore whether the resulting measurements were consistent with established differences in gait observed between individuals with and without a history of stroke. The observation of expected between-group differences provided additional evidence that the measurements derived from the pose estimation approach were capturing established gait characteristics. To better reflect this rationale, we have revised the Results (please see the section of red text below “Major comments, 9” under the heading *Why the secondary analyses?* for specific changes regarding this topic) and Discussion sections (please see the section of red text below) to clarify that this analysis was exploratory and intended to provide additional context regarding the validity of the measurements rather than address the primary study objective.

***“Interpretation of Differences Between Groups”***

*“The differences in gait kinematics between people with and without a history of stroke have been extensively described.(36,37) The results of our between-group comparison of gait parameters demonstrated that the methodology used in this study detected expected differences in gait kinematics as there were statistically significant differences in all gait parameters between groups in both settings. Although not a primary objective of the study, the observed between-group differences were consistent with previously reported gait differences between individuals with and without a history of stroke. This finding provides additional context regarding the clinical meaningfulness of the measurements derived from the pose estimation approach.”*

HOME VIDEO FEASIBILITY

Major comments, 11. The Substantiation of at-home measurement of gait kinematics states that all videos were processed with pose estimation, but this seems inaccurate since the authors report poor quality resulting in exclusion. Further if the authors aim to speak to the feasibility of capturing/analyzing video collected at home they should present data to assess this.

All submitted videos were processed through the pose estimation pipeline and analyzed via MATLAB; however, not all pose estimation outputs resulted in successful MATLAB analysis because of insufficient video quality and/or tracking errors. We have revised the Discussion section to clarify this distinction. Additionally, to better substantiate the feasibility of home-based video assessment, we have added data regarding the proportion of submitted videos that yielded analyzable gait data and the reasons for exclusion within the Discussion.

***Substantiation of At-Home Measurement of Gait Kinematics***

*“All video data reported in this study were processed using freely available pose estimation software(15) and a custom MATLAB(25) program. A total of 50 home videos were submitted, and 43 resulted in usable data (86%). The primary reasons for exclusion were poor video quality with visible pixelation and occasional failures of the pose estimation algorithm to reliably distinguish the participant from background objects and people. These findings demonstrate the feasibility of obtaining useful kinematic data with limited resources and the practicality of capturing real-world gait. The ability to directly measure gait in an individual’s natural environment opens new opportunities to evaluate mobility with greater ecological validity.”*

EDITORIAL REVISIONS

Minor comments, 1. I suggest revisiting the use of several terms to ensure clarity. For example, in the first paragraph (intro) gait dysfunction and gait impairment seem interchangeable, but as the manuscript progresses this isn’t the case. Performance is used in the third paragraph (intro) right after a distinction between capacity and performance is made.

We agree that some of the terminology used throughout the manuscript could be interpreted inconsistently. Thank you for bringing this to our attention. We have reviewed the manuscript and revised the language to improve clarity and consistency. Specifically, we have ensured consistently within the manuscript regarding “dysfunction” and “impairment, and we have revised our use of the terms “capacity” and “performance” to avoid confusion. We appreciate the opportunity to improve the manuscript's precision and readability.

Minor comments, 2. Citation 6 seems incorrect. Please check.

We carefully reviewed Citation 6 and did not identify an error. However, we added an additional supporting reference and corrected the manuscript reference formatting to comply with PLOS ONE requirements.
