# Supplementary material for: In-clinic versus at-home gait kinematics in adults with and without stroke: Associations across environments
Source: PLoS One. 2026 Nov 11;21(11):e0361481. doi: 10.1371/journal.pone.0361481 (PMC13754162; doi:10.1371/journal.pone.0361481)
Supplement: Peer review history

# Peer review: Decision Letter 0

**DOI:** 10.1371/journal.pone.0361481.r001

**Authors:** Anne E. Martin

3 Aug 2026

Dear Dr. Ahrns,

Thank you for submitting your manuscript to PLOS ONE. After careful consideration, we feel that it has merit but does not fully meet PLOS ONE’s publication criteria as it currently stands. Therefore, we invite you to submit a revised version of the manuscript that addresses the points raised during the review process.

Please address all reviewer comments, paying particular attention to Reviewer 2's comments.

Please submit your revised manuscript by Oct 02 2026 11:59PM. If you will need more time than this to complete your revisions, please reply to this message or contact the journal office at plosone@plos.org. When you're ready to submit your revision, log on to https://www.editorialmanager.com/pone/ and select the 'Submissions Needing Revision' folder to locate your manuscript file.

- A letter that responds to each point raised by the academic editor and reviewer(s). You should upload this letter as a separate file labeled 'Response to Reviewers'.
- A marked-up copy of your manuscript that highlights changes made to the original version. You should upload this as a separate file labeled 'Revised Manuscript with Track Changes'.
- An unmarked version of your revised paper without tracked changes. You should upload this as a separate file labeled 'Manuscript'.

If you would like to make changes to your financial disclosure, please include your updated statement in your cover letter. Guidelines for resubmitting your figure files are available below the reviewer comments at the end of this letter.

If applicable, we recommend that you deposit your laboratory protocols in protocols.io to enhance the reproducibility of your results. Protocols.io assigns your protocol its own identifier (DOI) so that it can be cited independently in the future. For instructions see: https://journals.plos.org/plosone/s/submission-guidelines#loc-laboratory-protocols. Additionally, PLOS ONE offers an option for publishing peer-reviewed Lab Protocol articles, which describe protocols hosted on protocols.io. Read more information on sharing protocols at https://plos.org/protocols?utm_medium=editorial-email&utm_source=authorletters&utm_campaign=protocols.

As the corresponding author, your ORCID iD is verified in the submission system and will appear in the published article. PLOS supports the use of ORCID, and we encourage all coauthors to register for an ORCID iD and use it as well. Please encourage your coauthors to verify their ORCID iD within the submission system before final acceptance, as unverified ORCID iDs will not appear in the published article. *Only*  the individual author can complete the verification step; PLOS staff *cannot*  verify ORCID iDs on behalf of authors.

We look forward to receiving your revised manuscript.

Kind regards,

Anne E. Martin

Academic Editor

PLOS One

Journal Requirements:

When submitting your revision, we need you to address these additional requirements.

1. Please ensure that your manuscript meets PLOS ONE's style requirements, including those for file naming. The PLOS ONE style templates can be found at

https://journals.plos.org/plosone/s/file?id=wjVg/PLOSOne_formatting_sample_main_body.pdf and

https://journals.plos.org/plosone/s/file?id=ba62/PLOSOne_formatting_sample_title_authors_affiliations.pdf

2. When completing the data availability statement of the submission form, you indicated that you will make your data available on acceptance. We strongly recommend all authors decide on a data sharing plan before acceptance, as the process can be lengthy and hold up publication timelines. Please note that, though access restrictions are acceptable now, your entire data will need to be made freely accessible if your manuscript is accepted for publication. This policy applies to all data except where public deposition would breach compliance with the protocol approved by your research ethics board. If you are unable to adhere to our open data policy, please kindly revise your statement to explain your reasoning and we will seek the editor's input on an exemption. Please be assured that, once you have provided your new statement, the assessment of your exemption will not hold up the peer review process.

3. If the reviewer comments include a recommendation to cite specific previously published works, please review and evaluate these publications to determine whether they are relevant and should be cited. There is no requirement to cite these works unless the editor has indicated otherwise.

[Note: HTML markup is below. Please do not edit.]

Reviewers' comments:

Reviewer's Responses to Questions

**Comments to the Author**

1. Is the manuscript technically sound, and do the data support the conclusions?

Reviewer #1: Yes

Reviewer #2: Partly

**********

2. Has the statistical analysis been performed appropriately and rigorously?

Reviewer #1: Yes

Reviewer #2: No

**********

3. Have the authors made all data underlying the findings in their manuscript fully available?

The PLOS Data policy

Reviewer #1: Yes

Reviewer #2: Yes

**********

4. Is the manuscript presented in an intelligible fashion and written in standard English?

Reviewer #1: Yes

Reviewer #2: Yes

**********

Reviewer #1: The research here reports a null finding with respect to differences between clinic and at-home gait kinematics in people with and without stroke. This investigation of the effects of changing the data-gathering environment for gait parameters is novel, and provides results that strengthen clinic-based gait measurement as estimating ‘true’ gait properties.

A significant part of the value of the current work is continuing to demonstrate to researchers involved with gait rehabilitation the power, efficiency, availability and low cost of Pose Estimation-based gait analysis. The paragraphs describing and supporting this method in the Introduction succinctly summarise supporting work published since the development of Pose Estimation.

However, there is a body of work that could support the logic for investigating the effects of environment change on gait parameters, and that is the work on Reinvestment conducted by Rich Masters and colleagues (Masters, R., & Maxwell, J. (2008). The theory of reinvestment. International Review of Sport and Exercise Psychology, 1(2), 160-183. Young, W. R., Olonilua, M., Masters, R. S., Dimitriadis, S., & Mark Williams, A. (2016). Examining links between anxiety, reinvestment and walking when talking by older adults during adaptive gait. Experimental brain research, 234(1), 161-172). This work highlights the possibility that gait measurement undertaken in clinics is assessing a consciously-controlled form of the target.

Movie directors, particularly those making Westerns, often struggled with actors whose walking gait became affected when shooting scenes. John Wayne was famously impatient with young actors who were unable to walk naturally on the movie set. Masters was one of the first researchers to call attention to the interfering effects of directing conscious attention to movement, terming the mechanism ‘reinvestment’.

So reinvestment was a real prospect for confounding in-clinic gait assessment, with the outcome being that the measures did not reflect the participants ‘real’ gait.

The results here indicate that reinvestment effects did not occur. The authors should add text taking credit for ruling out this possibility.

Reviewer #2: Major comments

1. The motivation for this work needs to be more clear. The second paragraph of the introduction discusses the discrepancy between capacity (i.e, if they can) and performance (i.e., if they do), but the majority of metrics in this work are at the impairment level. The connection between this discrepancy and measures of impairment needs to be clear if this is what motivates the work.

2. Justification for the hypotheses are needed. Why do the authors think how someone walks will be different just because they are at home vs in the lab. The conditions at home do not necessarily have to be different. For example, the floor in a lab/clinic is not very different than tile or hardwood floors that are often in homes. Additionally, if gait parameters are different is this necessarily bad? The introduction appears to couch differences in gait measurements in the lab vs in the community as a limitation/weakness to current measurement (paragraph 2 of the intro), but humans modify their gait in response to their environment regularly. I am unclear what the expected result would be given that there is no information about how similar/dissimilar the home environment is compared to the lab/clinic.

3. The Data Processing and Analysis section states that “up to four segments of the participant walking toward the camera” were analyzed. Is a segment a ‘walking pass?’ Does this mean that no data were analyzed when the participant was walking away from the camera? Conversely, “as many segments as possible” were analyzed if there were not 4 segments. Was there a minimum number of segments?

4. Each individual had multiple steps from which most of the gait metrics could be calculated. How was this handled in the analysis? Were all the steps averaged for each person to ensure independence of observations?

5. The number of subjects does not match the number of exclusions shown in Figure 3 or described in the results.

6. Shorter and longer step time/length were used instead of left/right. Was the same leg always the shorter/longer? How was it handled if this wasn’t the case? I assume this could have occurred in the healthy group.

7. I am unclear why the gait speeds presented in Table 3 are so different than the gait speeds in Table 2.

8. Figure 4 shows that there appears to be a fairly significant outliner in thr gorup of individuals with stroke on STA. A sensitivity analysis should be conducted to see how that individual is influencing the results.

9. I am unclear why the secondary analyses that were complete were done. Specifically, why were the gait metrics between groups compared? We know that individuals with stroke walk differently. Assessing the interaction between group and condition seems far more interesting and informative than the analyses done. I acknowledge that there is not a non parametric test of a 2x 2 mixed ANOVA; however, there are other options (robust ANOVA, GEE, etc) that should be conducted.

10. The summary of the conclusion at the beginning of the discussion is significantly oversimplified. Correlations range from weak to strong, so I am unclear how the findings support that “gait measured in the clinic provides meaningful insights into gait performance in daily life.”

11. The Substantiation of at-home measurement of gait kinematics states that all videos were processed with pose estimation, but this seems inaccurate since the authors report poor quality resulting in exclusion. Furhter if the authors aim to speak to the feasibility of capturing/analyzing video collected at home they should present data to assess this.

12. In the discussion, the author circle back to this distinction between capacity and performance. I remain unclear of how this work connects to that previous work as the authors are measuring an entirely different ICF domain (impairment) and they are measuring the same domain just in a different environment as opposed to 2 different domains.

13. In the discussion, the authors state that the between group difference support that PoseEstimation can detect expected differences in gait, but this is not a purpose of the work, so I again question the utility of this analysis.

Minor comments

1. I suggest revisiting the use of several terms to ensure clarity. For example, in the first paragraph (intro) gait dysfunction and gait impairment seem interchangeable, but as the manuscript progresses this isn’t the case. Performance is used in the third paragraph (intro) right after a distinction between capacity and performance is made.

2. Citation 6 seems incorrect. Please check.

3. I suggest stating what modified independent means in Table 2.

4. I suggest adding the time component of 48 hours to Figure 1.

5. Please include the assessments completed in the text rather than the supplemental materials.

6. Please provide a copy of the instructions provided to the participants as a supplemental.

7. Significance is not indicated in Table 4 the same between groups.

**********

what does this mean?). If published, this will include your full peer review and any attached files.

If you choose “no”, your identity will remain anonymous but your review may still be made public.

**Do you want your identity to be public for this peer review?**  For information about this choice, including consent withdrawal, please see our Privacy Policy

Reviewer #1: No

Reviewer #2: No

**********

[NOTE: If reviewer comments were submitted as an attachment file, they will be attached to this email and accessible via the submission site. Please log into your account, locate the manuscript record, and check for the action link "View Attachments". If this link does not appear, there are no attachment files.]

To ensure your figures meet our technical requirements, please review our figure guidelines: https://journals.plos.org/plosone/s/figures

You may also use PLOS’s free figure tool, NAAS, to help you prepare publication quality figures: https://journals.plos.org/plosone/s/figures#loc-tools-for-figure-preparation.

NAAS will assess whether your figures meet our technical requirements by comparing each figure against our figure specifications.

# Author comment: Author response to Decision Letter 1

**DOI:** 10.1371/journal.pone.0361481.r002

4 Sep 2026

Please see attached Response to Reviewers document where we responded to specific reviewer and editor comments. Thank you for this opportunity.

Attachment Submitted filename: PONE-D-26-29985R1_Response-to-Reviewers.docx

# Peer review: Decision Letter 1

**DOI:** 10.1371/journal.pone.0361481.r003

**Authors:** Anne E. Martin

13 Oct 2026

In-Clinic Versus At-Home Gait Kinematics in Adults With and Without Stroke: Associations Across Environments

PONE-D-26-29985R1

Dear Dr. Ahrns,

We’re pleased to inform you that your manuscript has been judged scientifically suitable for publication and will be formally accepted for publication once it meets all outstanding technical requirements.

Within one week, you’ll receive an e-mail detailing the required amendments. When these have been addressed, you’ll receive a formal acceptance letter and your manuscript will be scheduled for publication.

An invoice will be generated when your article is formally accepted. Please note, if your institution has a publishing partnership with PLOS and your article meets the relevant criteria, all or part of your publication costs will be covered. Please make sure your user information is up-to-date by logging into Editorial Manager at Editorial Manager® and clicking the ‘Update My Information' link at the top of the page. For questions related to billing, please contact billing support.

If your institution or institutions have a press office, please notify them about your upcoming paper to help maximize its impact. If they’ll be preparing press materials, please inform our press team as soon as possible -- no later than 48 hours after receiving the formal acceptance. Your manuscript will remain under strict press embargo until 2 pm Eastern Time on the date of publication. For more information, please contact onepress@plos.org.

Kind regards,

Anne E. Martin

Academic Editor

PLOS One

Additional Editor Comments (optional):

Reviewers' comments:

Reviewer's Responses to Questions

**Comments to the Author**

Reviewer #2: (No Response)

**********

2. Is the manuscript technically sound, and do the data support the conclusions?

Reviewer #2: Yes

**********

3. Has the statistical analysis been performed appropriately and rigorously?

Reviewer #2: Yes

**********

4. Have the authors made all data underlying the findings in their manuscript fully available?

The PLOS Data policy

Reviewer #2: Yes

**********

5. Is the manuscript presented in an intelligible fashion and written in standard English?

Reviewer #2: Yes

**********

Reviewer #2: A majority of my comments have been addresses; however my original major comment 6 (Was the same leg always the shorter/longer? How was it handled if this wasn’t the case? I assume this could have occurred in the healthy group.) was not fully addressed. I was asking if the same leg always had the shorter/longer step length for the same person. For individuals with stroke, I imagine the same leg had the shorter/longer step length consistently, but I'm less confident that this was the case for neurologically intact individuals. How was it handled if the shorter and longer step switched withing the same person.

**********

what does this mean?). If published, this will include your full peer review and any attached files.

If you choose “no”, your identity will remain anonymous but your review may still be made public.

**Do you want your identity to be public for this peer review?**  For information about this choice, including consent withdrawal, please see our Privacy Policy

Reviewer #2: **Yes:**  Margaret French

**********

# Editor report: Acceptance letter

**DOI:** 10.1371/journal.pone.0361481.r004

**Authors:** Anne E. Martin

PONE-D-26-29985R1

PLOS One

Dear Dr. Ahrns,

I'm pleased to inform you that your manuscript has been deemed suitable for publication in PLOS One. Congratulations! Your manuscript is now being handed over to our production team.

At this stage, our production department will prepare your paper for publication. This includes ensuring the following:

* All references, tables, and figures are properly cited

* All relevant supporting information is included in the manuscript submission,

* There are no issues that prevent the paper from being properly typeset

You will receive further instructions from the production team, including instructions on how to review your proof when it is ready. Please keep in mind that we are working through a large volume of accepted articles, so please give us a few days to review your paper and let you know the next and final steps.

Lastly, if your institution or institutions have a press office, please let them know about your upcoming paper now to help maximize its impact. If they'll be preparing press materials, please inform our press team within the next 48 hours. Your manuscript will remain under strict press embargo until 2 pm Eastern Time on the date of publication. For more information, please contact onepress@plos.org.

You will receive an invoice from PLOS for your publication fee after your manuscript has reached the completed accept phase. If you receive an email requesting payment before acceptance or for any other service, this may be a phishing scheme. Learn how to identify phishing emails and protect your accounts at https://explore.plos.org/phishing.

If we can help with anything else, please email us at customercare@plos.org.

Thank you for submitting your work to PLOS One and supporting open access.

Kind regards,

PLOS One Editorial Office Staff

on behalf of

Dr. Anne E. Martin

Academic Editor

PLOS One
